# Higher PEEP reduces duration of mechanical ventilation in neonatal respiratory distress syndrome of late preterm and term newborn infants

**DOI:** 10.1038/s41598-025-24335-7

**Published:** 2025-11-18

**Authors:** Tao Ning, Qiong Xue, Pingli Wang, Xudong Zhang, Xiaoxue Shan, Liuqing Yang, Mei Yuan, Chang Dong, Song Liu, Suyue Zhu, Huaiyu Yan

**Affiliations:** Department of Pediatrics, The Affiliated Suqian Hospital of Xuzhou Medical University, 138 Huanghe Road, Sucheng District, Suqian, 223800 Jiangsu China

**Keywords:** Late preterm infant, Term infant, Neonatal respiratory distress syndrome, Positive end expiratory pressure, Mechanical ventilation, Oxygenation index, Diseases, Health care, Medical research

## Abstract

Mechanical ventilation was frequently conducted in late preterm and term newborn infants because of their severity of neonatal respiratory distress syndrome (NRDS), but the level of positive end expiratory pressure (PEEP) used was not explicit. This study aimed to investigate the efficacy and safety of higher-PEEP in the treatment of NRDS in these infants. Initially, 80 newborn late preterm and term infants diagnosed with NRDS were enrolled, a total of 26 infants were excluded because they were not within the gestational age range of 34^+ 0^ to 39^+ 6^ weeks or did not receive mechanical ventilation. Of 54 eligible infants, 6 were excluded: 3 for pre-existing pneumothorax before mechanical ventilation, 1 for hospital transfer, 1 for withdrawal of treatment, and 1 for misdiagnosis with transient tachypnea. Ultimately, 48 infants remained. Following a simple randomization procedure, 23 were assigned to higher-PEEP group and 25 to the control group. The duration of mechanical ventilation was regarded as the primary outcome. We also collected and analyzed data of other clinical factors. We found that higher-PEEP group had significantly shorter durations of mechanical ventilation (*P* = 0.008) and oxygen inhalation (*P* = 0.002) compared to the control. Additionally, the fraction of inspired oxygen (FiO_2_) (*P* = 0.001) and oxygenation index (OI) (*P* = 0.048) at 24 h after birth were lower in higher-PEEP group compared to the control. Furthermore, higher-PEEP group had a shorter duration of hospitalization (*P* = 0.033). However, no significant differences were observed in the comparisons of complications between the two groups. In summary, higher PEEP could reduce the duration of mechanical ventilation by preserving adequate functional residual capacity, without increasing rates of adverse effects.

## Introduction

NRDS was a common disease in preterm infants, primarily due to lung immaturity and insufficient pulmonary surfactants. The late preterm infants (born at gestational age of 34 to 36^+ 6^ weeks) and term infants still had certain morbidity, mainly due to prematurity, gestational diabetes mellitus and cesarean section, and frequently presented with more severe conditions, requiring mechanical ventilation and repeat surfactants. Traditional mechanical ventilation protocols were effective but might cause lung injury. Some scholars observed that pediatricians tended to use low levels of PEEP and inherently accepted higher FiO_2_, although such practices might lead to worse outcomes^1^. In the treatment of mechanical ventilation in NRDS, peak inspiratory pressure (PIP) and FiO_2_ were preferentially increased because of small tidal volume (VT) and impaired oxygenation function, but the optimal PEEP remained uncertain.

The commonly used levels for PEEP were 3–9 cmH_2_O^2^. Some clinical evidences suggested that PEEP levels ≥ 5 cmH_2_O during mechanical ventilation might be beneficial by preserving functional residual capacity (FRC) and preventing lung collapse^3^. PEEP levels were dynamically adjusted between 6 and 8 cmH_2_O in clinical practice. But we observed that the levels of PEEP at 6–8 cmH_2_O often failed to maintain early stabilization in late preterm and term newborn infants needing mechanical ventilation, some cases exhibiting rapid deterioration, necessitating higher PIP and FiO_2_ to compensate for inadequate gas exchange. This compensatory strategy, however, risked exacerbating alveolar hyperoxia injury, barotrauma, and atelectrauma (cyclic collapse-reopening injury).

Given that PEEP primarily served to maintain FRC, we hypothesized that there might be a critical PEEP threshold that effectively maintained lung inflation during the expiratory phase in mechanically ventilated NRDS cases with markedly reduced FRC, without harming the immature lung. Clinical evidence showed that higher PEEP had already been adopted in delivery room resuscitation and post-extubation non-invasive respiratory support^4^. So, we conducted the present study to evaluate the efficacy and safety of higher PEEP levels in late preterm infants and term infants.

## Results

**Fig. 1:**
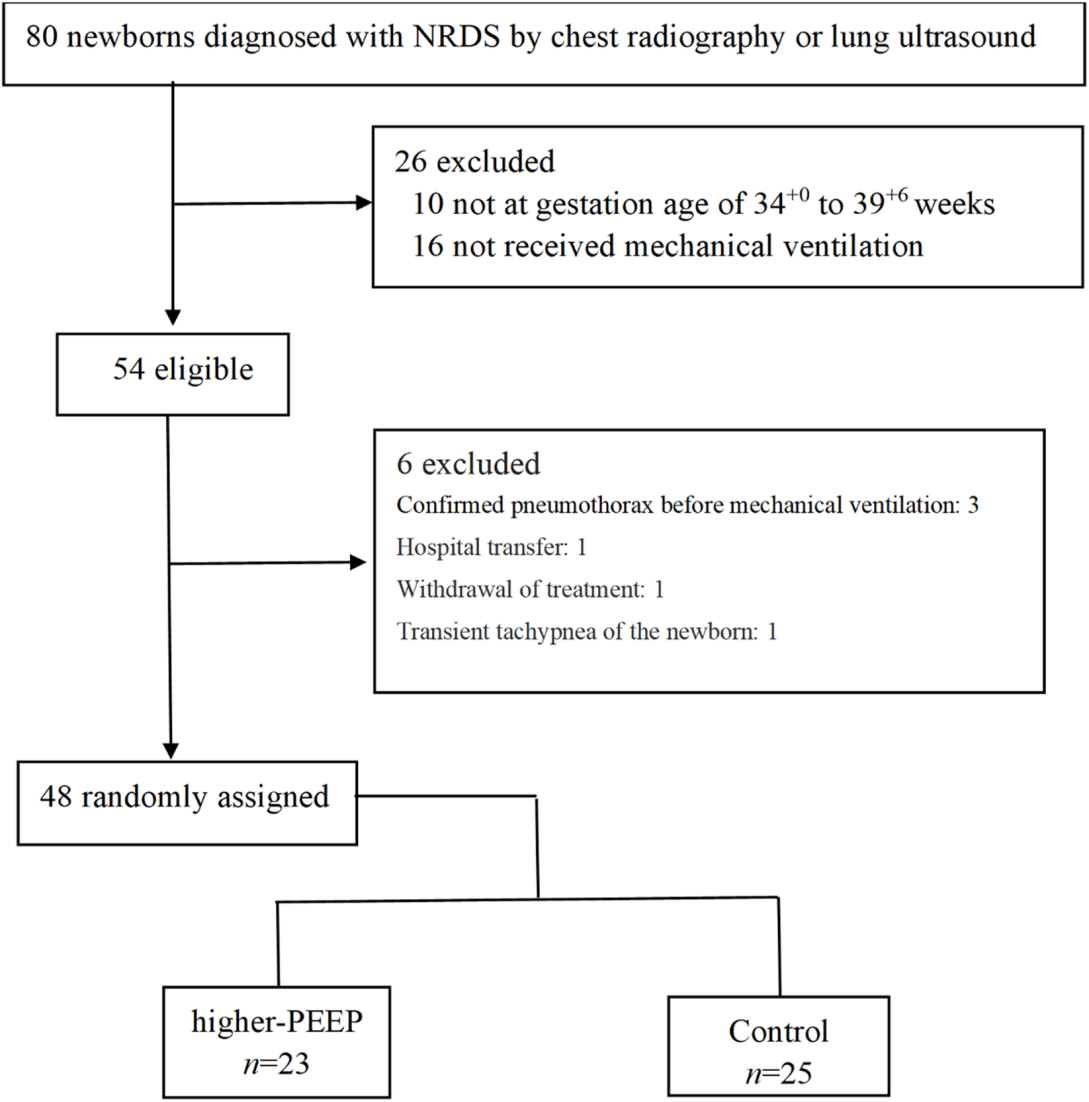
CONSORT flow chart for the enrolled late preterm and term newborn infants.

Initially, 80 newborn late preterm and term infants diagnosed with NRDS were enrolled, a total of 26 infants were excluded because they were not within the gestational age range of 34^+ 0^ to 39^+ 6^ weeks or did not receive mechanical ventilation. All parents of the remaining 54 infants provided written informed consent before the allocation. Of 54 eligible infants, 6 were excluded: 3 for pre-existing pneumothorax before mechanical ventilation, 1 for hospital transfer, 1 for withdrawal of treatment, and 1 for misdiagnosis with transient tachypnea. Ultimately, 48 infants remained. Following a simple randomization procedure, 23 were assigned to higher-PEEP group and 25 to the control group. Twins or multiples were individually randomized (Fig. 1). All these late preterm and term newborn infants developed respiratory distress within 6 to 12 h after birth and were enrolled within 12 h after birth.

A comparison between the two groups showed no difference in the perinatal factors and demographic characteristics of the late preterm and term infants, shown in Table 1. The SNAPPE-II score (Score for Neonatal Acute Physiology with Perinatal Extension II) assessed disease severity by scoring six physiological parameters based on their most abnormal values during the first 12 h after admission. The six components were mean blood pressure, temperature, ratio of arterial oxygen partial pressure to fractional inspired oxygen (PaO_2_/ FiO_2_ ratio), serum pH, urine output, multiple seizures. These components provided a quantitative measure of the infant’s acute physiological stability. As shown in Table 2, there was no difference in SNAPPE-II score between the two groups (*P* = 0.983). Upon comparing PEEP, PIP, MAP, FiO_2_, and OI between the two groups after initiation of mechanical ventilation (i.e., prior to PEEP adjustment), no significant intergroup differences were observed.

**Table 1:** Perinatal factors and demographic characteristics of late preterm and term newborn infants.

Perinatal factors and demographic characteristics	Higher-PEEP (*n* = 23)	Control (*n* = 25)	*p* value
Mother age, mean (SD), year	31.3 (4.62)	31.6 (3.76)	0.808
Antenatal steroid use, *n* (%)	0	0	1.000
Gestational diabetes mellitus, *n* (%)	5 (21.7)	5 (20.0)	1.000
Cesarean section, *n* (%)	18 (78.3)	22 (88.0)	0.454
Prenatal antibiotic duration, mean (SD), day	0.6 (0.82)	1.0 (1.97)	0.359
Male gender, *n* (%)	18 (78.3)	17 (68.0)	0.523
Gestational age, mean (SD), week	36.4 (2.13)	36.5(1.55)	0.905
Birth weight, mean (SD), g	2773 (596)	2895 (485)	0.445
Apgar score at 5 min, mean (SD)	9.3 (0.75)	8.8 (1.45)	0.145

Data were expressed as number (%) where Pearson’s chi-square test or Fisher’s exact test was used or as mean (SD) where Student’s independent sample *t* test was used for comparison.

Abbreviations: SD, standard deviation.

**Table 2:** Clinical data of late preterm and term newborn infants.

Clinical data	Higher-PEEP (*n* = 23)	Control (*n* = 25)	*p* value
Age at randomization, mean (SD), hour	4.61 (2.15)	4.40 (2.02)	0.730
SNAPPE-II score, mean (SD)	40.9 (12.67)	40.8 (9.2)	0.983
Breast feeding, *n* (%)	6 (26.1)	8 (32.0)	0.653
Pulmonary surfactant, mean (SD), mg/kg	98.9 (43.92)	103.2 (40.22)	0.738
Invasive ventilation duration, mean (SD), hour	60.2 (21.03)	83.1(34.44)	0.008
Non-invasive ventilation duration, mean (SD), hour	56.4 (29.12)	62.8 (53.91)	0.608
Oxygen inhalation duration, mean (SD), hour	0	11.24 (16.57)	0.002
Hospitalization duration, mean (SD), day	9.8 (2.20)	12.5 (5.60)	0.033

Data were expressed as number (%) where Pearson’s chi-square test or Fisher’s exact test was used or as mean (SD) where Student’s independent sample *t* test was used for comparison.

The SNAPPE-II score was calculated based on the most abnormal values (i.e., the worst values) recorded during the first 12 h after neonatal admission.

Abbreviations: SD, standard deviation.

In the control group, three neonates were switched to high-frequency oscillatory ventilation (HFOV) due to worsening status. Their durations of conventional mechanical ventilation were 16, 18, and 22 h, respectively. After transitioning to HFOV, both MAP and FiO_2_ were increased compared to previous settings and maintained at higher parameters beyond 24 h.

### Reduced duration of mechanical ventilation in higher-PEEP group

**Fig. 2:**
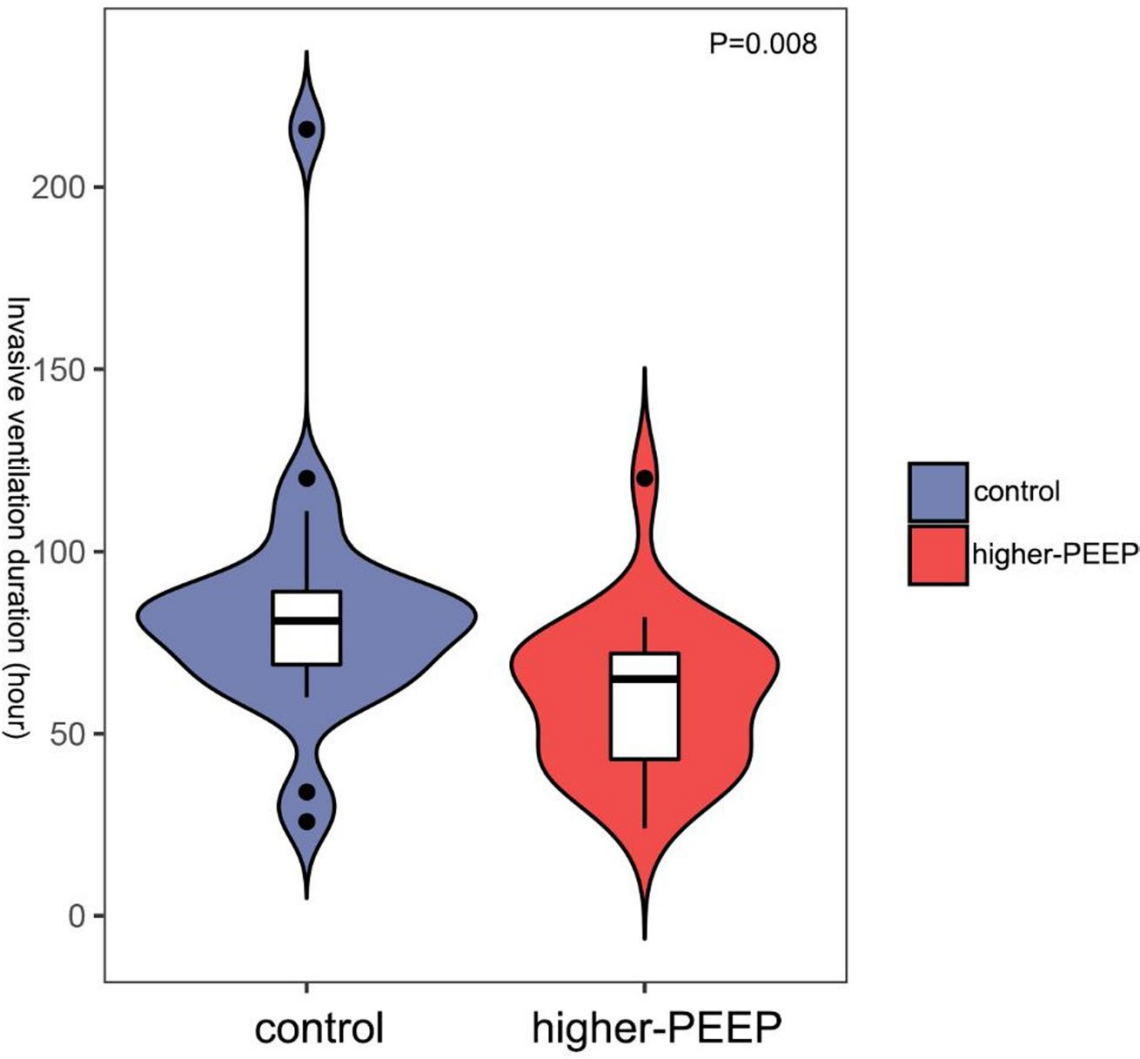
Higher-PEEP group had a shorter duration of mechanical ventilation compared to the control (*P* = 0.008).

As shown in Fig. 2; Table 2, higher-PEEP group had significantly shorter duration of mechanical ventilation compared to the control (*P* = 0.008). However, there was no significant difference in the duration of non-invasive ventilation between two groups (*P* = 0.608). Further analysis revealed that the duration of oxygen inhalation also differed significantly (*P* = 0.002), consistent with the observed differences in mechanical ventilation duration.

### Increased PEEP, PIP and mean airway pressure (MAP) in higher-PEEP group

**Fig. 3:**
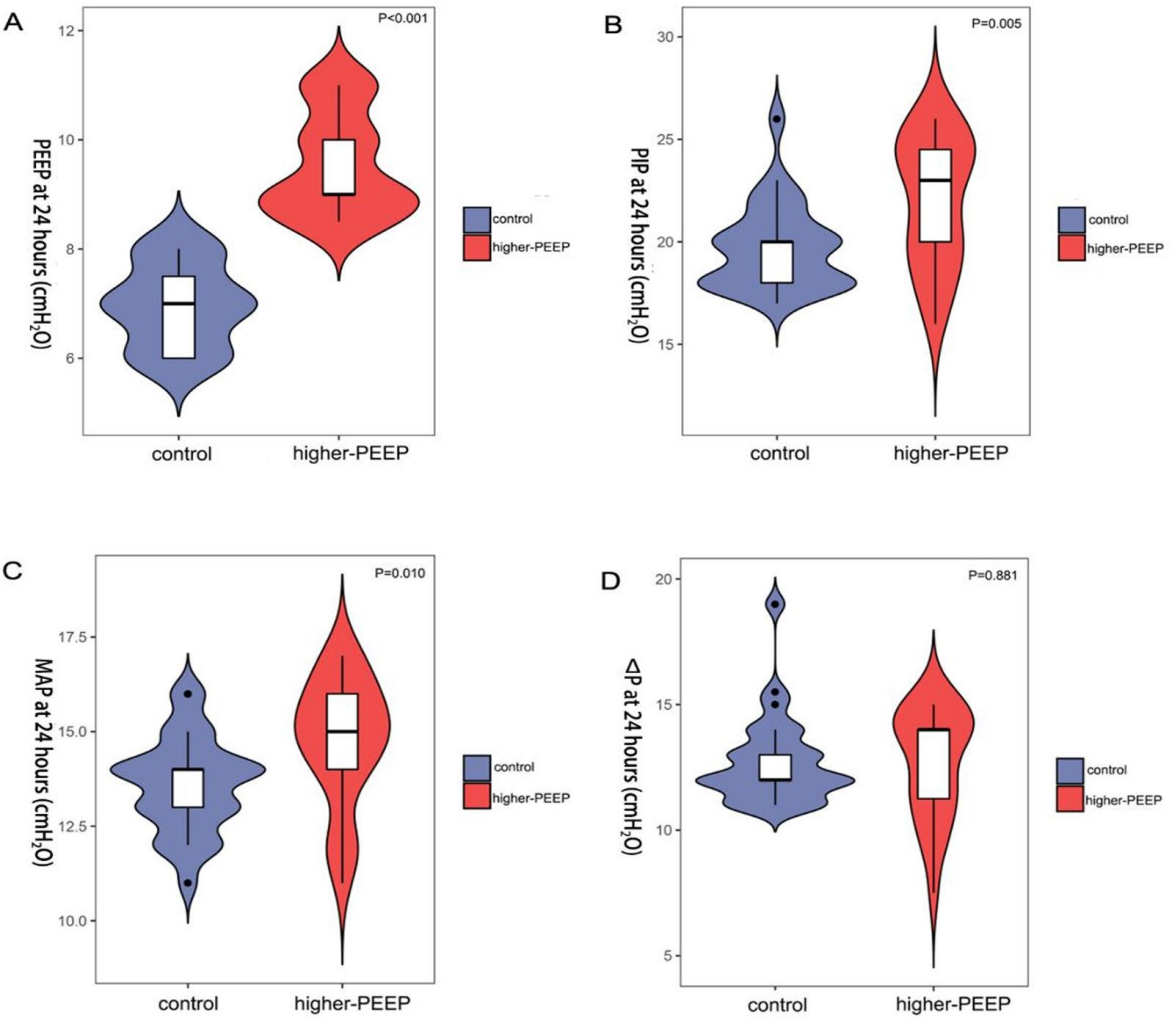
During mechanical ventilation, higher-PEEP group exhibited significantly higher PEEP (*P* < 0.001), PIP (*P* = 0.005), and MAP (*P* = 0.010) at 24 h after birth compared to the control (**A**–**C**); but ΔP (*P* = 0.881) was not significantly different (**D**).

During the mechanical ventilation, higher-PEEP group exhibited significantly higher PEEP (*P* < 0.001), PIP (*P* = 0.005), unsurprisingly, and MAP (*P* = 0.010) at 24 h after birth during the clinical climax compared to the control (Fig. 3A-C); but the ΔP (driving pressure, PIP minus PEEP) was not significantly different (*P* = 0.881; Fig. 3D).

### Reduced FiO_2_ and OI in higher-PEEP group

**Fig. 4:**
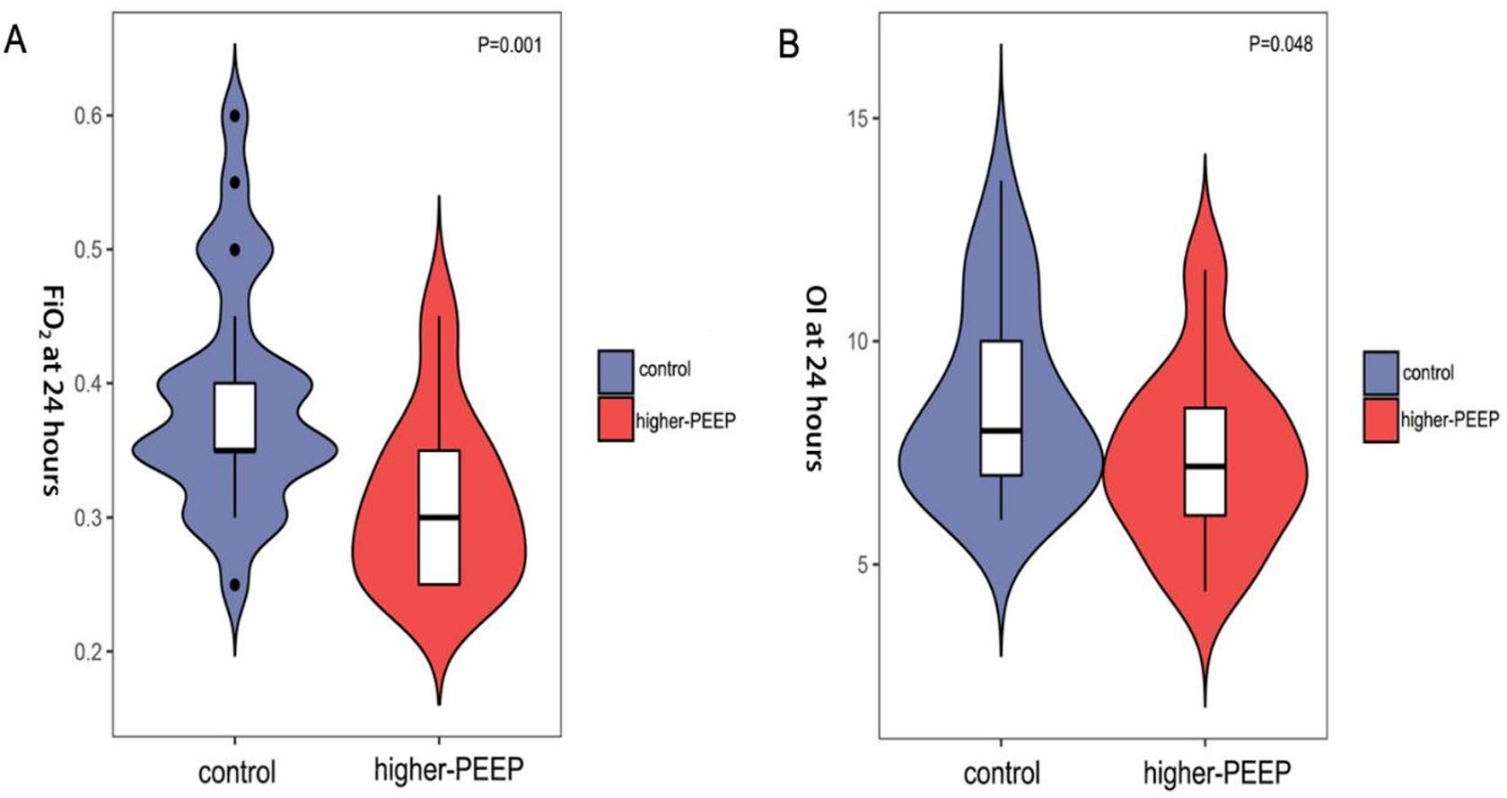
During mechanical ventilation, the FiO_2_ (*P* = 0.001), OI (*P* = 0.048) at 24 h after birth were markedly lower in higher-PEEP group (**A**,**B**).

Interestingly, the FiO_2_ at 24 h after birth was markedly lower in higher-PEEP group (*P* = 0.001; Fig. 4A). When comparing the OI at 24 h after birth, we observed that higher-PEEP group had lower OI than the control (*P* = 0.048; Fig. 4B), suggesting improved oxygenation efficiency.

### Reduced duration of hospitalization in higher-PEEP group

**Fig. 5:**
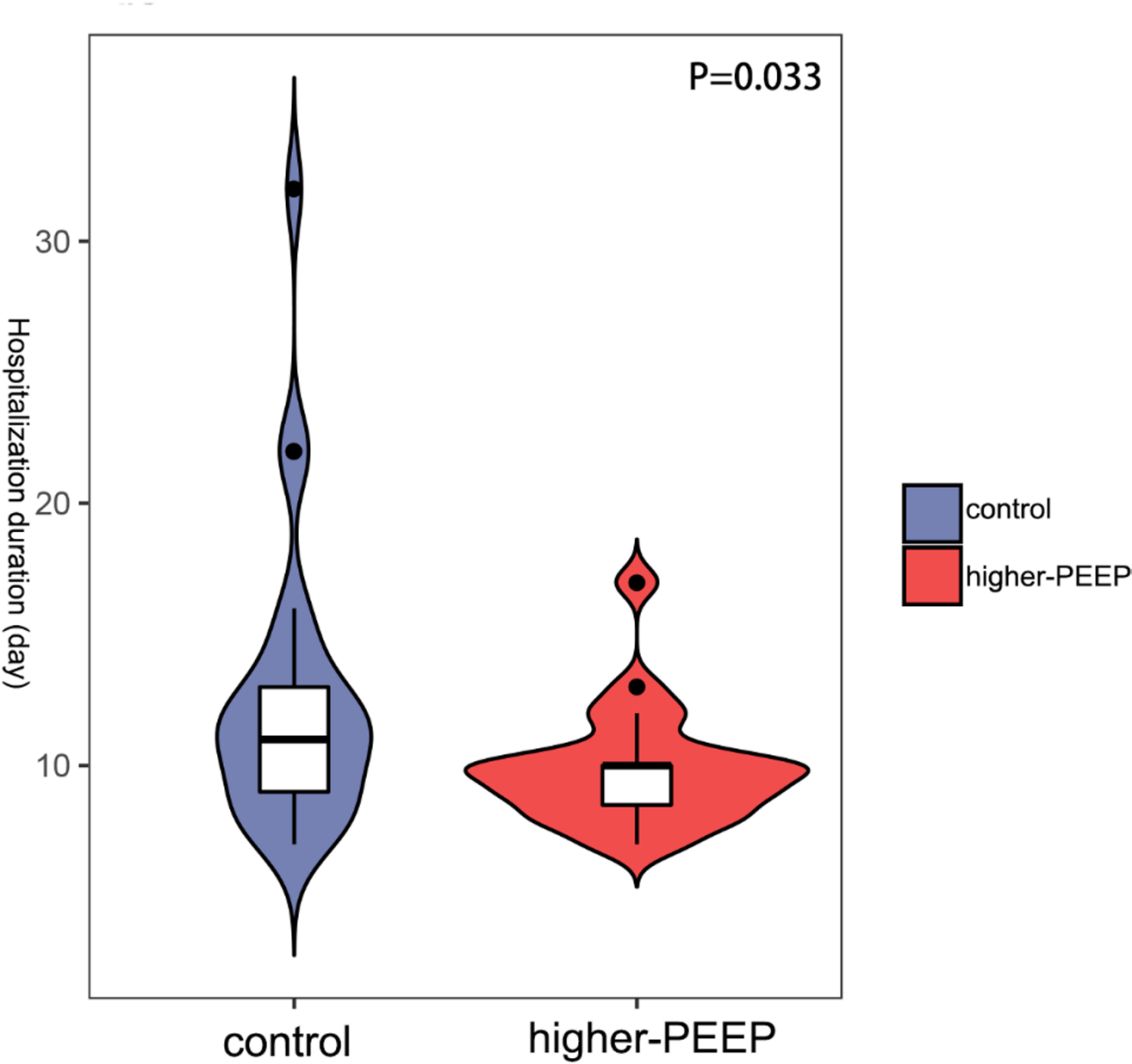
The duration of hospitalization (*P* = 0.033) was shorter in higher-PEEP group.

Furthermore, the duration of hospitalization was shorter in higher-PEEP group (*P* = 0.033; Fig. 5).

### Comparisons of complications between two groups

We observed the clinical outcomes of both groups and found that higher-PEEP group exhibited fewer cases of moderate to severe pulmonary hypertension, though this difference was not statistically significant (*P* = 0.292). In higher-PEEP group, three cases of moderate to severe pulmonary hypertension were identified by routine bedside echocardiography. None of these cases presented with a significant upper-lower limb oxygen saturation gradient, and their OI values did not reach the threshold for initiating vasoactive drug therapy. In the control group, seven cases of moderate to severe pulmonary hypertension were observed; one of these progressed to persistent pulmonary hypertension of the newborn and required treatment with inhaled nitric oxide, while the other six did not receive vasoactive drug therapy. Pulmonary hemorrhage occurred in one infant in the control, while no case was observed in higher-PEEP group. Both groups had one case of pneumothorax each; however, higher-PEEP group’s case was mild and required no intervention, whereas the control group’s case necessitated chest tube drainage. Encouragingly, no intraventricular hemorrhage (IVH) occurred in higher-PEEP group, while the control had three cases of Grade I-II IVH, though this difference also lacked statistical significance. Furthermore, neither group experienced periventricular leukomalacia (PVL), retinopathy of prematurity (ROP), hemodynamically significant patent ductus arteriosus (hsPDA), neonatal necrotizing enterocolitis (NEC), late-onset sepsis, and ventilator-associated pneumonia (VAP) (Table 3).

**Table 3:** Clinical outcomes of late preterm and term newborn infants.

Clinical outcomes	Higher-PEEP (*n* = 23)	Control (*n* = 25)	*p* value
Pulmonary hemorrhage, *n* (%)	0	1 (4.0)	1.000
Pneumothorax, *n* (%)	1 (4.3)	1 (4.0)	1.000
Atelectasis, *n* (%)	0	1 (4.0)	1.000
Moderate and severe pulmonary hypertension*, *n* (%)	3 (13.0)	7 (28.0)	0.292
Persistent pulmonary hypertension of the newborn, *n* (%)	0	1^#^ (4.0)	1.000
Switch to high-frequency oscillatory ventilation, *n* (%)	0	3 (12.0)	0.235
Intraventricular hemorrhage, *n* (%)	Grade I–II: 0 Grade III–IV: 0	Grade I–II: 3 (12.0) Grade III–IV: 0	0.235 1.000

Data were expressed as number (%) where Pearson’s chi-square test or Fisher’s exact test was used.

*Pulmonary artery pressure exceeded half of the systemic arterial systolic pressure.

^#^Require treatment with inhaled nitric oxide.

## Discussion

NRDS was a common disease in newborn infants, often requiring respiratory support due to its severity, posing significant challenges for neonatologists. Regarding the severe cases, conventional mechanical ventilation combined with surfactant therapy remained the most widely used approach. Modern ventilation strategies emphasized not only gas exchange efficacy but also lung protection.

The NRDS of late preterm and term newborn infants often required invasive mechanical ventilation and repeated doses of surfactants. These infants often exhibited better respiratory muscle compensation, the early diagnosis and treatment of NRDS in this population tended to occur later compared to preterm infants of lower gestational ages. By the time symptoms worsened, significant pulmonary collapse and exudation were often already evident, with a higher incidence of pulmonary hypertension or pneumothorax^5^, posing significant challenges in respiratory support. Moreover, or neonates born via cesarean section who develop NRDS, lung exudation was often very severe. The plasma proteins exuded into the alveoli further inhibit pulmonary surfactant activity. The duration of pulmonary surfactant efficacy might be shorter in these cases, potentially necessitating multiple doses^6^.

In this study, according to the treatment protocol of local clinicians, corticosteroids such as dexamethasone or betamethasone were not routinely administered prenatally for neonates delivered after 34 weeks’ gestation, as the use of antenatal steroids beyond 34 weeks of gestation remained controversial^7^. Additionally, the high cesarean section rates (78.3% in higher-PEEP group and 88.0% in the control) might further increase the risk of NRDS. We speculated that the development of NRDS was mainly due to prematurity, gestational diabetes mellitus and cesarean section.

Although it was widely recognized that optimal PEEP, lower VT, and reduced PIP were essential for improving ventilation and minimizing lung injury, there was still no consensus on the ideal PEEP level for late preterm and term infants. During the management of NRDS in late preterm and term infants, we also found that the PEEP levels at 6–8 cmH_2_O were frequently proved inadequate, necessitating higher PIP or FiO_2_ to achieve sufficient oxygenation, which could also cause lung injury^8^. To better maintain FRC, we increased PEEP levels to 8.5–11 cmH_2_O in clinical practice and evaluated the efficacy and safety of higher PEEP levels. Our primary outcome was the duration of mechanical ventilation, which showed a significant decrease in higher-PEEP group. Other improvements were also observed in outcomes such as the shorter duration of oxygen inhalation and hospitalization, and lower OI and FiO_2_. at 24 h after birth during the clinical climax compared to the control. However, no significant differences were observed in the comparisons of complications between the two groups.

Ultimately, 48 infants remained, 23 were assigned to higher-PEEP group and 25 to the control group. All these late preterm and term newborn infants developed respiratory distress within 6 to 12 h after birth and were enrolled within 12 h after birth. We compared the SNAPPE-II score and found no difference between the two groups. Upon comparing PEEP, PIP, MAP, FiO_2_, and OI between the two groups after initiation of mechanical ventilation (i.e., prior to PEEP adjustment), no significant intergroup differences were observed. The lack of statistical significance in these parameters was expected, as the initial ventilator settings for infants in both groups were highly similar at the time of enrollment, as defined in our intervention protocols.

During mechanical ventilation, PEEP served as a continuous airflow that helped to maintain alveolar stability and FRC, stabilize the chest wall and upper airway, decrease pulmonary resistance and reduce alveolar edema, thus improving VT and oxygenation, minimizing atelectasis, and reducing obstructive apnea^9^. When PEEP levels were inadequate, insufficient FRC and oxygenation could conduct persistent alveolar exudation, edema, hypoxia, and acidosis, further damaging pulmonary capillaries and leading to clinical complications, including persistent pulmonary hypertension of the newborn, gastrointestinal disorder, and cerebral injury^10^.

Given that the key pathophysiological mechanisms underlying NRDS progression were suboptimal FRC and reduced lung compliance, optimal PEEP was considered as the primary parameter of ventilator, which could also enhance alveolar gas reserve while expanding the exchange area between the respiratory membrane and capillaries, thereby prolonging gas exchange duration and opportunities, and it thinned the respiratory membrane and counteracted capillary exudation, ultimately enhancing ventilation efficiency and oxygenation. We considered whether a higher PEEP could be adopted to maintain near-physiological FRC, thereby reducing dependence on other parameters. In clinical practice, we routinely analyzed the inflection point on the pressure-volume curve to determine optimal PEEP. Notably, in severe NRDS cases characterized by markedly flattened P-V loops, the lower inflection point consistently demonstrated a rightward shift (higher pressure range), indicating increased alveolar recruitment pressure requirements. But the dentification of optimal PEEP levels was difficult.

Clinically, when using non-invasive ventilation to treat NRDS, the PEEP was often set higher than 8 cmH_2_O^11^. Another study in preterm lambs demonstrated that dynamic PEEP levels of 14–20 cmH_2_O in mechanical ventilation minimized lung injury compared to lower PEEP. Moreover, higher PEEP caused minimal cardiopulmonary interference, likely due to the use of lower ΔP at the same VT^12^. One study showed that sustained inflation and dynamic PEEP up to 15 cmH2O significantly decreased the intubation, surfactant administration in the delivery room, mechanical ventilation and its mean duration < 72 h of life, administration of a 2nd dose of surfactant, postnatal corticosteroids, and the rate of bronchopulmonary dysplasia^13^. Results from animal studies further suggested that using higher PEEP levels (8–12 cmH2O) in mechanical ventilation improved gas exchange and avoided lung collapse when compared to lower levels of PEEP^14^. This was precisely why we investigated the applicability of higher PEEP levels (8.5–11 cmH_2_O) in NRDS management.

In this study, we set PEEP levels at 6–8 cmH_2_O in the control and 8.5–11 cmH_2_O in higher-PEEP group, respectively. The lower limit of 8.5 cmH_2_O for higher-PEEP group was chosen because some patients did not require excessively high PEEP according to the clinical status. Both groups maintained their respective PEEP levels for at least 24 h, ensuring that all late preterm and term newborn infants received adequate pressure support before reaching the clinical climax of NRDS. Higher-PEEP group exhibited significantly higher PEEP, PIP, unsurprisingly, and MAP at 24 h after birth during the clinical climax compared to the control. The promising findings in this research were that we have identified not only significantly shorter duration of mechanical ventilation and oxygen inhalation, but also lower levels of FiO_2_, and OI in higher-PEEP group. Furthermore, the duration of hospitalization was shorter in higher-PEEP group, further supporting the potential benefits of higher PEEP ventilation strategy. Scholars also demonstrated that the use of higher MAP was safe and there was no more airleak^15^.

For newborn infants, the use of higher PEEP levels should be approached with caution due to the risk of air leak. However, we found that the control group developed one pneumothorax case necessitating closed thoracic drainage, whereas higher-PEEP group had only one mild pneumothorax case managed conservatively without intervention. Air leak primarily resulted from excessive alveolar volume, which occurred mainly due to pulmonary hyperinflation or uneven gas distribution^10^. Their FRC and lung compliance improved during treatment but never fully normalized to physiological levels despite receiving higher PEEP and surfactants. Consequently, their FRC never exceeded the normal range during the expiratory phase. Additionally, none of the late preterm and term newborn infants achieved excessive VT because of our strict VT control (4–6 ml/kg). When addressing uneven ventilation, we needed to focus the gas flow between the well-aerated alveoli and the collapsed alveoli.

As we all know, the greater the FRC of alveoli, the higher their compliance and the lower the distending pressure required during inspiration. In contrast, collapsed alveoli demanded substantially greater pressure to generate equivalent VT. Under the same inspiratory ΔP, alveoli with bette FRC tended to expand more easily, while those with poor FRC collapsed, leading to uneven ventilation, thus consequent atelectasis and hyperinflation. During this process, the delivered VT was consistently inadequate, necessitating increased respiratory effort by the infants to compensate. This compensatory mechanism further resulted in alveolar overdistension in regions with better FRC, ultimately precipitating air leak^16^. These could explain our clinical observations. In our study, we excluded three newborn infants because they had already developed pneumothorax prior to mechanical ventilation, who either received no PEEP or inadequate PEEP during non-invasive ventilation.

Conversely, optimal PEEP could recruit more alveoli for ventilation, thereby reducing atelectasis and pulmonary hyperinflation, which to some extent mitigated uneven alveolar gas distribution. Surfactant also exhibited comparable therapeutic effects, it was proved that surfactant use was associated with a significant decrease in risk of air leak^17^. In our study, all cases were treated with surfactants at the initiation of mechanical ventilation, and there was no difference in the dosage of surfactant between two groups. In a study investigating the optimal PEEP selection for non-invasive ventilation after extubation in extremely preterm infants, researchers found that higher PEEP (9–11 cmH_2_O) reduced the reintubation rate within a week post-extubation without increasing pneumothorax and gastrointestinal perforation^4^.

The above pathophysiological mechanisms indicated that higher-PEEP group would be expected to demonstrate a reduced ΔP for a given VT compared to the control group. But we noticed that there was no significant difference in ΔP between the two groups, which might be related to the clinicians’ practice. Our target VT range was 4–6 cmH_2_O rather than a specific fixed value. As was well known, VT was directly related to ΔP. In a trial of preterm lambs, while maintaining a constant PEEP levels, the 30 cmH_2_O ΔP strategy resulted in quicker aeration but higher VT (> 8 ml/kg) and lung injury compared with ΔP 20 cmH_2_O group; and the single PEEP without tidal inflation group resulted in the least lung injury^8^. These findings suggested that high ΔP and the resulting large VT were the main causes of lung injury. In a large-scale clinical randomized trial of 3562 patients with acute respiratory distress syndrome, researchers frequently employed PEEP settings higher than 10 cmH_2_O, they found that ΔP was the ventilation variable that best stratified risk. Decreases in ΔP owing to changes in ventilator settings were strongly associated with increased survival, and reducing ΔP and increasing PEEP was associated with increased survival^18^. Some scholars also demonstrated that limiting ΔP and mechanical power required reducing VT and increasing PEEP, decreasing lung injury^1^. In an experimental animal study, researchers found that the gene expression of markers of early lung injury were inversely related to magnitude of PEEP, being lowest in the higher PEEP group (20 cmH_2_O dynamic); PEEP levels had no impact on lung injury in the dependent lung^19^. Although our higher-PEEP group did not demonstrate a significant reduction in ΔP compared to the control group, the mere increase in PEEP levels without a corresponding rise in ΔP did not exacerbate lung injury.

We also observed no significant differences in the comparison of clinical complications between the two groups, suggesting that higher PEEP maintained favorable FRC without compromising hemodynamics. We observed that higher-PEEP group exhibited fewer cases of moderate to severe pulmonary hypertension, though this difference was not statistically significant. There was no case of pulmonary hemorrhage in higher-PEEP group, but that occurred in one infant in the control. Encouragingly, no IVH occurred in higher-PEEP group, while the control had three cases of Grade I-II IVH, though this difference also lacked statistical significance.

Notably, in the control group, three infants were switched to HFOV by the attending physicians for safety reasons due to disease progression when their MAP already exceeded 15 cmH_2_O. Their durations of conventional mechanical ventilation were 16, 18, and 22 h, respectively. After transitioning to HFOV, both MAP and FiO_2_ were increased compared to previous settings and maintained at higher parameters beyond 24 h. As a standard practice, the MAP during HFOV was typically higher than in conventional ventilation, yielding superior clinical outcomes. Some scholars argued that the primary advantage of high-frequency ventilation laid in their ability to maintain a higher MAP, which promoted alveolar recruitment and stabilization throughout the respiratory cycle^20^. Similarly, in conventional ventilation, increasing PEEP could achieve comparable effects by reopening collapsed alveoli, mainly the poorly-aerated alveoli, left lung and gravity-dependent lung regions^8^. Despite reclassifying their data under the control group for analysis, this adjustment inherently narrowed the observed differences when compared with higher-PEEP group.

In summary, we have observed the safety and efficacy of 8.5–11 cmH_2_O PEEP levels in treating NRDS of late preterm and term newborn infants, with no increased incidence of the complications, providing evidence for future clinical practice. Higher-PEEP ventilation might emerge as a novel therapeutic strategy, mitigating ventilator-associated complications. Further researches were needed to establish evidence-based PEEP guidelines for the vulnerable population.

As the limitations of our study: (1) this was not a multi-center study, and the sample size was small, thus limiting the statistical power of our results; (2) the management of NRDS and the adjustment of ventilator parameters were closely influenced by the individual experience of local physicians; (3) no bronchoalveolar lavage studies were performed, precluding direct measurement of alveolar injury; (4) no genetic research data related to pulmonary injury and metabolism were available. As a pilot study, our next step will be to enlarge the study cohort and further investigate the genomics and metabolomics in higher-PEEP group.

## Methods

### Study design and participants

This study was a prospective, randomized controlled trial conducted in level III NICU of The Affiliated Suqian Hospital of Xuzhou Medical University from April 2025 to August 2025.

When these late preterm and term newborn infants developed respiratory distress within 6 to 12 h after birth, we performed both radiographic and lung ultrasound examinations on them in the NICU. The inclusion criteria were: (1) NRDS diagnosed by chest radiography or lung ultrasound; (2) at gestation age of 34^+ 0^ to 39^+ 6^ weeks; (3) requirement of mechanical ventilation: ① requiring FiO_2_ > 0.4 on non-invasive ventilation; ② persistent or worsening respiratory acidosis, typically defined as: pH < 7.20–7.25, PaCO_2_ > 60–65 mmHg (8.0–8.7 kPa); ③ increased work of breathing with significant symptoms of respiratory distress despite optimal non-invasive support; ④ recurrent apnea or respiratory pauses requiring ventilation with bag and mask. The exclusion criteria were: (1) chromosomal abnormalities; (2) congenital malformations affecting respiratory function; (3) history of severe adverse perinatal conditions, such as intrauterine distress, severe birth asphyxia, severe intrauterine infection, congenital pneumonia, and meconium aspiration syndrome (gestational diabetes mellitus excluded); (4) pneumothorax occurring prior to mechanical ventilation; (5) missing of guardian informed consent.

### Intervention protocols

#### Control group

**Initial ventilation mode and parameter settings**: synchronized intermittent mandatory ventilation (SIMV) + pressure support ventilation (PSV); FiO_2_: adjusted dynamically to maintain target arterial oxygen saturation (SaO_2_) between 91% and 95% (measured via right wrist probe); inspiratory time: 0.35–0.4 s; respiratory rate: 30–50 breaths/min; VT: 4.0–6.0 ml/kg; PIP: adjusted to maintain VT and target arterial carbon dioxide partial pressure (PaCO_2_) between 35 and 55 mmHg; PEEP: adjustable within 6–8 cmH_2_O for ≥ 24 h, after 24 h, PEEP could be adjusted within the assigned range or reduced at clinician discretion. Clinicians were aware of group assignments and aimed to wean ventilator early.

**Extubation criteria**: spontaneous breathing with adequate chest rise and resolved respiratory distress; normal arterial blood gas values; MAP ≤ 8 cmH_2_O; FiO_2_ ≤ 30%; respiratory rate ≤ 20 breaths/min.

**Post-extubation support**: nasal continuous positive airway pressure (PEEP ≤ 6 cmH_2_O), hood oxygen therapy (FiO_2_ ≤ 30%), or room air.

**Reintubation**: Performed if extubation failed (criteria identical to initial intubation indications), with PEEP adjusted based on clinical status.

#### Higher-PEEP group

**Initial ventilation mode and parameter settings**: SIMV + PSV; FiO_2_: adjusted dynamically to maintain target SaO_2_ between 91% and 95% (measured via right wrist probe); inspiratory time: 0.35–0.4 s; respiratory rate: 30–50 breaths/min; VT: 4.0–6.0 ml/kg; PIP: adjusted to maintain VT and target PaCO_2_ between 35 and 55 mmHg; PEEP: initial setting at 7 cmH_2_O, gradually increased to 9 cmH_2_O over 2 h (to avoid rapid lung inflation injury), adjusted between 8.5 and 11 cmH_2_O according to lung inflation confirmed via radiographic examination, clinician-adjusted, and maintained for ≥ 24 h; after 24 h, PEEP could be adjusted within the assigned range or reduced at clinician discretion. Clinicians were aware of group assignments and aimed to wean ventilator early.

**Extubation criteria**: identical to the control group.

**Post-Extubation support**: identical to the control group.

**Reintubation**: identical to the control group.

### Surfactant application strategy

For NRDS requiring mechanical ventilation, the surfactant was administered immediately after endotracheal intubation. The surfactant in this study was calf pulmonary surfactant (a bovine surfactant product marketed as Kelisu in China). The initial dose was administered at a range of 70 to 100 mg/kg. If there was no significant improvement after the initial dose or if the condition worsened again after initial improvement, a reassessment was conducted. Based on clinical manifestations and lung imaging, if the NRDS was still judged to be severe, repeat surfactant administration was considered, with a dosage equal to the initial dose. The interval between doses was generally 6 to 12 h.

### Equipment and adjuncts

All endotracheal intubations were performed via the oral route. The invasive ventilators were Dräger V500 or V300 (Drägerwerk AG & Co. KGaA, Lübeck, Germany). The non-invasive ventilators were Comen NV8 (Shenzhen Comen Medical Instruments Co., Ltd., Guangdong Province, China), and the size of nasal prong was determined by infant weight. Routine phenobarbital sodium was treated during intubation, and additional phenobarbital sodium or midazolam was used for patient-ventilator asynchrony. No caffeine citrate was used. The administration of repeated dose of surfactant was based on persistent clinical, radiological, and symptomatic evidence of NRDS.

### Outcomes

The primary outcome was the duration of mechanical ventilation (hour). We also collected the data of OI at 24 h after birth during the clinical climax, calculated as (MAP×FiO_2_ × 100)/PaO_2_, and cumulative dosage of surfactant (mg/kg). Furthermore, the other ventilation parameters, non-invasive ventilation duration, oxygen hood therapy duration, clinical complications, and length of hospitalization were collected and analyzed.

### Experimental deviation

In the control group, three infants were switched to HFOV by the attending physicians for safety reasons due to disease progression when their MAP already exceeded 15 cmH_2_O. Their durations of conventional mechanical ventilation were 16, 18, and 22 h, respectively. After transitioning to HFOV, both MAP and FiO_2_ were increased compared to previous settings and maintained at higher parameters beyond 24 h; and the dose of surfactant they received showed no significant difference compared to other infants whose MAP exceeded 15 cmH_2_O.

### Data analysis

All analyses were performed based on the intention-to-treat principle, with preterm infants retained in their originally assigned groups for all outcome assessments. Clinical data from both groups were analyzed according to case report forms. Collected data underwent completeness checks to address any gaps, followed by dual independent entry into SPSS 26 software. Post-entry, all datasets were subjected to logical validation. Continuous variables were analyzed using parametric tests (*t*-test) or non-parametric tests (Wilcoxon rank-sum test), while categorical variables were assessed via Pearson’s chi-square or Fisher’s exact tests. These methods were employed to compare intergroup differences, therapeutic efficacy, and safety outcomes. The ethics committee of The Affiliated Suqian Hospital of Xuzhou Medical University conducted independent safety analyses for preterm infants, and all statistical evaluations were prospectively planned prior to data interrogation. This trial was registered with the Chinese Clinical Trial Registry on 20/08/2025, number ChiCTR2500107900.

## Data Availability

The raw data supporting the findings of this study are available from the corresponding author on reasonable request.
